# Molecular Dynamic Simulations of Bromodomain and Extra-Terminal Protein 4 Bonded to Potent Inhibitors

**DOI:** 10.3390/molecules27010118

**Published:** 2021-12-26

**Authors:** Siao Chen, Yi He, Yajiao Geng, Zhi Wang, Lu Han, Weiwei Han

**Affiliations:** Key Laboratory for Molecular Enzymology and Engineering of Ministry of Education, National Engineering Laboratory for AIDS Vaccine, School of Life Science, Jilin University, Changchun 130012, China; sachen20@mails.jlu.edu.cn (S.C.); heyi21@mails.jlu.edu.cn (Y.H.); gengyj21@mails.jlu.edu.cn (Y.G.); wangzhi@jlu.edu.cn (Z.W.)

**Keywords:** bromodomain and extra-terminal protein 4 (BRD4), inhibitors, molecular dynamic simulations, conformational changes, MM-GBSA calculations

## Abstract

Bromodomain and extra-terminal domain (BET) subfamily is the most studied subfamily of bromodomain-containing proteins (BCPs) family which can modulate acetylation signal transduction and produce diverse physiological functions. Thus, the BET family can be treated as an alternative strategy for targeting androgen-receptor (AR)-driven cancers. In order to explore the effect of inhibitors binding to BRD4 (the most studied member of BET family), four 150 ns molecular dynamic simulations were performed (free BRD4, Cpd4-BRD4, Cpd9-BRD4 and Cpd19-BRD4). Docking studies showed that Cpd9 and Cpd19 were located at the active pocket, as well as Cpd4. Molecular dynamics (MD) simulations indicated that only Cpd19 binding to BRD4 can induce residue Trp81-Ala89 partly become α-helix during MD simulations. MM-GBSA calculations suggested that Cpd19 had the best binding effect with BRD4 followed by Cpd4 and Cpd9. Computational alanine scanning results indicated that mutations in Phe83 made the greatest effects in Cpd9-BRD4 and Cpd19-BRD4 complexes, showing that Phe83 may play crucial roles in Cpd9 and Cpd19 binding to BRD4. Our results can provide some useful clues for further BCPs family search.

## 1. Introduction

**Figure 1:**
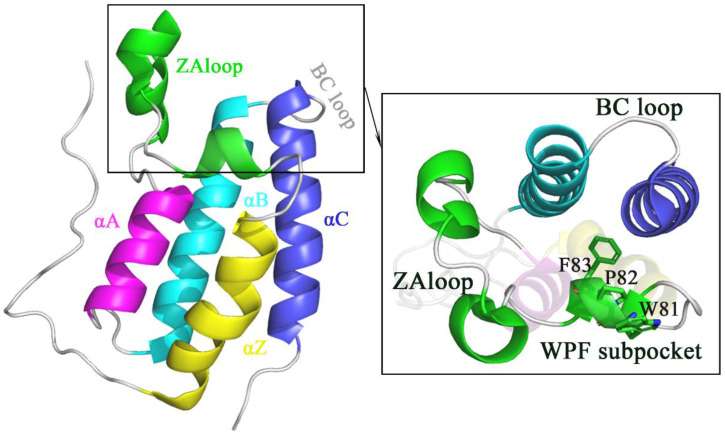
The structure of BRD4 protein and the binding pocket of BRD4.

Bromodomain-containing proteins (BCPs) can decipher the acetylation code of proteins via binding to their acetylated lysine (KAc) residues, which further modulates acetylation signal transduction and produces diverse physiological functions [1,2,3,4,5,6,7,8,9,10]. BCPs are divided into eight subfamilies containing the bromodomain and extra-terminal domain (BET) subfamily [11,12]. The BET subfamily [12] consists of BRD2 [13], BRD3 [14], BRD4 [12,15] and BRD testis-specific protein BRDT [16,17]. The BET protein family is highly similar in structure and function and is mainly composed of BD1, BD2 and ET domains and CHAT and CTM functional domains. BD1 and BD2 are responsible for the recognition and binding of histone acetylation sites at the H3 and H4 ends. For BRD4 protein, BD1 and BD2 also have partial kinase properties. BRD4-BD1 protein consists of A, B, C, Z four α-helix, ZA and BC two loops, in addition to ZA channel, WPF pocket and the cavity between four α-helix. The active pocket of BRD4 was presented in Figure 1. Recognition of histones with BRD4 is mediated through these cavities. Androgen regulated BRDs increased chromatin accessibility by enhancing AR recruitment to chromatin, which may drive cancer progression. BET inhibitors could be an alternative strategy for targeting AR-driven cancers due to the interaction between the BET family proteins and AR. In particular, the effect of BRD inhibitors is focused on cell growth and resistance in cancer cells and on inflammatory signaling in immune cells [18]. The use of such inhibitors has helped to understand the cell/tissue-specific functions that these proteins perform.

Many BRD inhibitors, such as (+)-JQ-1 [19], OTX-015 [20], I-BET762 [21], CPI0610 [22] and ABBV-07 [23] have been exploited. For example, Hu et al. pointed out some novel BRD4-selective inhibitors with an adihydroquinoxalin-2(1H)-one scaffold from the PLK1-BRD4 inhibitor [24,25]. Watts et al. designed a series of ALK and BRD4 dual inhibitors from BI-2536 [26]. Liu et al. described a series of compounds with different selectivity between PLK1 and BRD4 using BI-2536 as the starting compound [27] and so on.

In 2019, Hu et al. performed further lead optimization to generate a novel potent series of bromodomain and extra-terminal (BET) inhibitors with a (1,2,4-triazol-5-yl)-3,4-dihydroquinoxalin-2(1H)-one structure, and they found that compound 19 is a potential BET protein drug candidate for the treatment of cancer [12]. However, what effects do the potent and selective inhibitors binding to BRD4 have on the active site? MD simulations were a widely used powerful tool, simulating the physical movements of atoms and molecules by Newton’s equations of motion and qualitatively and quantitatively analyze conformational changes related to its function. Fernando D. Prieto-Martínez et al. performed the MD simulations to explore the conformational changes for Flavonoids binding to BRD4. The results showed that amentoflavone makes numerous contacts in the ZA channel [28]. In order to explore the effect of inhibitors binding to BRD4, four 150 ns molecular dynamic simulations were performed (free BRD4, compound 4 (Cpd4)-BRD4, compound 9 (Cpd9)-BRD4, compound 19 (Cpd19)-BRD4). Our theoretical results may be useful for designing excellent inhibitors for BRD4.

## 2. Methods

### 2.1. Protein Prepare

BRD4 was obtained from the Protein Data Bank (https://www.rcsb.org, accessed on 20 Febraury 2019) and used as starting structures (PDB code: 6JI3) [12]. The 3D structure of BRD4-Cpd4 was download from PDB (PDB code: 6JI4) [12]. The 3D structure of Cpd9 and Cpd19 were obtained by Gaussian View [29] and then optimized with Gaussian 09 software [29] at B3LYP 6-31G* set [30]. Then, Cpd9 and Cpd19 were docked to BRD4 with AutoDock 4.2.6 software [31]. The Lamarckian genetic algorithm was used to predict the stereo conformation and select the lowest energy system for further MD.

### 2.2. Molecular Dynamics Simulations

The complexes of the three inhibitors and BRD4 were performed by Amber 16 software with 150 ns molecular dynamics simulations (The sufficiency to really reach the proper equilibrium of the MD simulation needs to be explored according to the characteristics of the system. We have found these papers for the BET family with MD simulations for 100–200 ns [32,33,34,35,36,37,38]. In this study, the 150 ns MD simulations were performed). The system setting and the 2D chemical structure of the potent inhibitors were shown in Table 1. The force field of BRD4 was ff99SB force field [39] and the inhibitors were generated with GAFF parameters and RESP partial charges through the Antechamber program. 

The protein-ligand complexes were placed in a cube box with a boundary of 12 Å filled with periodic boundary conditions and a TIP3P water model [40]. Cl^−^ was added to maintain the electrical neutrality of the system [41]. The energy of the system was minimized by the steepest descent method and conjugate gradient algorithm, and then the simulated system was heated (from 0–310 K), density balanced (50 ps), and slowly released (500 ps at 310 K), respectively.

At last, four 150 ns MD unconstrained simulations were conducted by Amber 16 packages using the particle-mesh Ewald (PME) method in each system [42]. The SHAKE algorithm is used to constrain the hydrogen bonds [43]. Langevin dynamics method (1 atm constant pressure and 310 K temperature) was treated as Langevin thermostat during MD simulations [44]. The Berendsen pressurizer was used for constant pressure simulation and the cut-off radius was set to 8 Å. All of the coordinates were saved every 2 ps. We have performed three times MD simulations for four systems (see Figures S1–S3).

In order to analyze the conformational changes of BRD4-inhibitors during simulations, its backbone was superimposed and then clustered with a distance cut-off of 2.5 Å. Root-mean-square deviation (RMSD), root-mean-square fluctuation (RMSF), hydrogen bonding, dihedral angle distribution, and secondary structure form action were analyzed by using the Cpptraj module in Amber 16. Moreover, the Bio3D software was employed to perform principal component fluctuation analysis and dynamic residue cross-correlation analysis. The popular VMD software was used to visualize the MD trajectories.

### 2.3. Principal Components Analysis (PCA) and Cross-Correlation Matrix 

PCA is a popular statistical multivariate method that analyzes trajectories data from MD simulations [45]. Dynamics cross-correlation matrix (DCCM) among the atoms of protein I and j (c_ij_) was selected as a matrix from MD trajectories using the Bio3d package by R program [46]. 

### 2.4. MM/GBSA Calculations

Free energy calculations play an important role in computational biology, such as drug design and protein structure prediction, which require the participation of free energy calculations of ligands binding to protein [47,48]. There are many ways to calculate the free energy of ligands binding, such as free energy perturbation, thermodynamic integration and so on. These methods are strict in theory, they require very high computation, and become very expensive and time-consuming with the increase of system size and have poor convergence for complex systems. However, MM/GBSA is an analytical technique widely used in MD simulations calculated by the MMPBSA.py program in the Amber 16 package using Generalized Born (GB) implicit solvent models [49,50]. The sum and the free energy are divided into the molecular mechanics and solvation energies, the energy of the acceptor, the Ligand and the complex in the solution were calculated. Then the difference energy was calculated, and the combined free energy data and energy decomposition data were obtained. A total of 1000 frames were extracted from the 150 ns in each MD simulation for the calculation of binding free energies.

## 3. Result and Discussion

### 3.1. Docking Study

The 3D structure of the complex Cpd4-BRD4 was downloaded from Protein Data Bank (PDB code 6JI3) [12]. Figure S4 indicated that the active residues around Cpd4 binding to BRD4. It can be seen that Asn140, Tyr97, Ile146, Phe83, Leu94, Pro82, Leu92 and Tyr139. In particular, Asn140 made a hydrogen bond with BRD4, which suggested that Asn140 may play important role in Cpd4 binding to BRD4.

**Figure 2:**
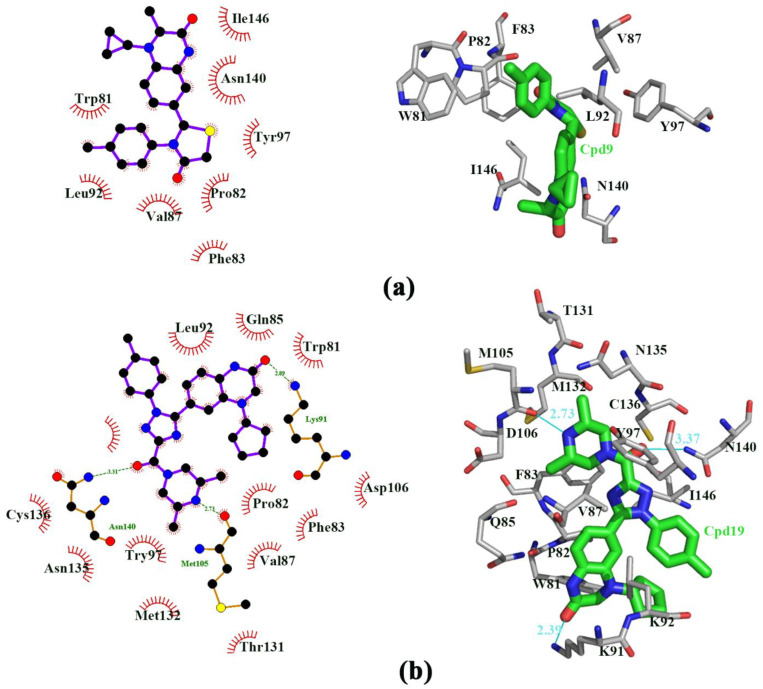
The binding pocket of (**a**) the active residues around Cpd9 binding to BRD4. (**b**) the active residues around Cpd19 binding to BRD4.

We redocked Cpd4 to BRD4 with AutoDock 4.2.6 software to compare with the docking pose with Cpd4 to BRD4 [30]. The best docking score was shown in Figure S5. The RMSD plot between the docking score and the reference Cpd4 was 0.88Å, showing that AutoDock 4.2.6 software was perfect and can be used for further docking study. Figure 2a,b showed that Cpd9 and Cpd19 were located at the active pocket. As seen in Figure 2a, Ile146, Asn140, Tyr97, Pro82, Phe83, Val87, Leu92 and Trp81 were the key residues for Cpd9 binding to BRD4. As seen from Figure 2b, Thr131, Asn135, Cys136, Met132, Met105, Asp136 Asn140, Tyr97, Ile146, Phe83, Val87, Pro82, Gln85, Trp81 and Lys91 were the key residues for Cpd19 binding to BRD4. In particular, Cys136, Met105 and Lys91 made hydrogen bonds with Cpd19. We also estimated the free energies of binding for BRD4-Cpd4, BRD4-Cpd9 and BRD4-Cpd19, which were −5.37, −5.80 and −7.16 kcal/mol, respectively. To sum up, the three complexes were all located at the active pocket and all were stable; thence the three complexes can be used for further study.

### 3.2. The Stability of the Four Systems

**Figure 3:**
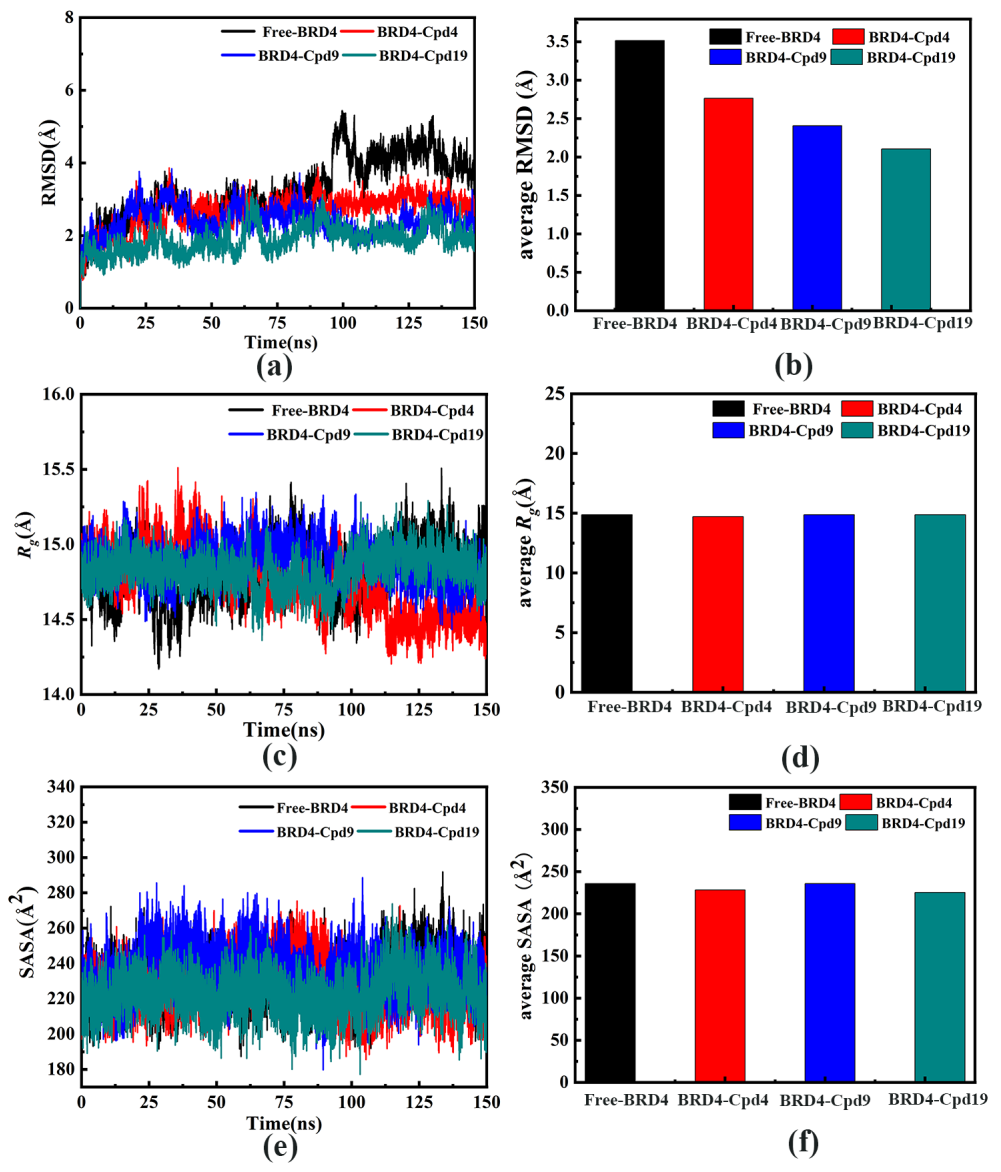
(**a**) Root-mean-square deviation (RMSD) for the backbone atoms, (**b**) the average RMSD of the four systems, (**c**) Radius-of-gyration (*R_g_*) plot and (**d**) the average *R_g_* plot, (**e**) Solvent-accessible-surface-area (SASA) plot (**f**) SASA average plot. The free-BRD4 is represented in black, BRD4-Cpd4 is represented in red, BRD4-Cpd9 is represented in blue and BRD4-Cpd19 is represented in green.

The RMSD plots during the 150 ns MD simulations were shown in Figure 3a,b. As seen in the RMSD plots, the three systems have reached equilibrium after 120 ns. Average RMSD values of free-BRD4, BRD4-Cpd4, BRD4-Cpd9, BRD4-Cpd19 were RMSD 3.51, 2.76, 2.41 and 2.11 Å, respectively. 

As shown in Figure 3c,d, the *R_g_* values of Free-BRD4, BRD4-Cpd4 and BRD4-Cpd9 were stabilized at 14.88 Å, 14.71 Å and 14.87 Å, respectively, while the BRD4-Cpd19 system was stabilized at 14.87 Å. Figure 3e,f showed the hydrophilicity of the four systems throughout the 150 ns MD simulations. In Figure 3e,f, it is obvious that the solvent accessible surface area (SASA) plot of free-BRD4 was similar to those of the other three systems in the 150 ns. All the results indicated that the four systems were stable and can be used for further study.

### 3.3. The Conformational Changes for Three Inhibitors Binding

**Figure 4:**
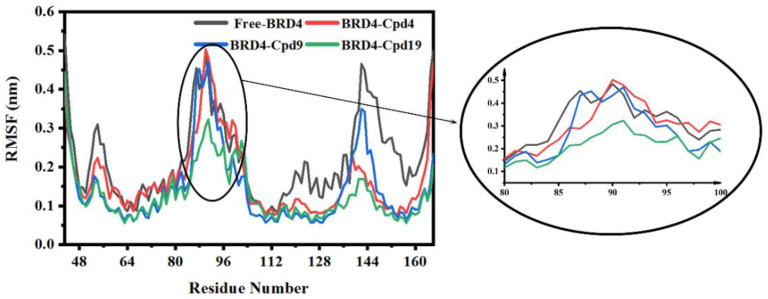
RMSF for the backbone atoms of the four systems. The free-BRD4 is represented in black, BRD4-Cpd4 is represented in red, BRD4-Cpd9 is represented in blue and BRD4-Cpd19 is represented in green.

After 150 ns MD simulations, the RMSF of the backbone atoms of the proteins in the four systems was calculated and the results were shown in Figure 4. It can be seen that residues 81 to 89 only in the Cpd19-BRD4 complex exhibited distinct atom-positional fluctuation amplitudes during 150 ns MD simulations. The results indicated that Cpd19 bound to BRD4 can make it less flexible and maintain an ordered structure of BRD4, thereby allowing the tighter fitting of Cpd19 into the enzyme active site.

**Figure 5:**
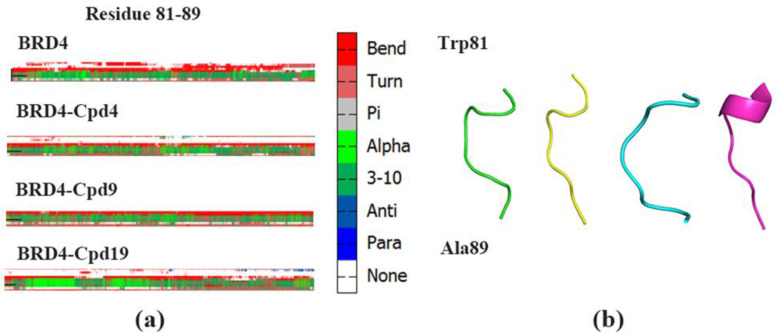
(**a**) Differences in the secondary structures of residues Trp81 to Ala89 of the four systems (**b**) Structure of Trp81 to Ala89.

To investigate the conformational changes, the difference in the secondary structure (DSSP) plot (Figure 5) was obtained. The DSSP of residue 81 to 83 in the BRD4-Cpd19 system significantly differed from the other three systems. As shown in Figure 5b, residue 81 to 83 in the BRD4-Cpd19 system partly formed α-helix. However, these residues of the other three systems showed a turn of BRD4. It was reported that the WPF shelf (W81, P82, F83), as well as the N140, are essential for acetylated lysine binding [43]. Our results also indicated that WPF shelves were important for inhibitors binding.

We calculated the probability to form α-helix of the four systems. The probabilities of residues Trp81, ProP82 and Phe83 in four systems were listed in Table 2. The probabilities in BRD4-Cpd19 (33.25 %) were considerably higher than those in the three other systems, suggesting that residue 81 to 83 in the BRD4-Cpd19 system partly formed α-helix during MD simulations. Therefore, the binding of Cpd19 was beneficial to maintain the order of the structure of BRD4 and it is useful to Cpd19 slide into the active pocket. Cpd19 is an excellent inhibitor binding to BRD4, which is consistent with the experimental data (IC_50_: 5.3 ± 0.4 nM) [12].

### 3.4. Cross-Correlation Analysis

**Figure 6:**
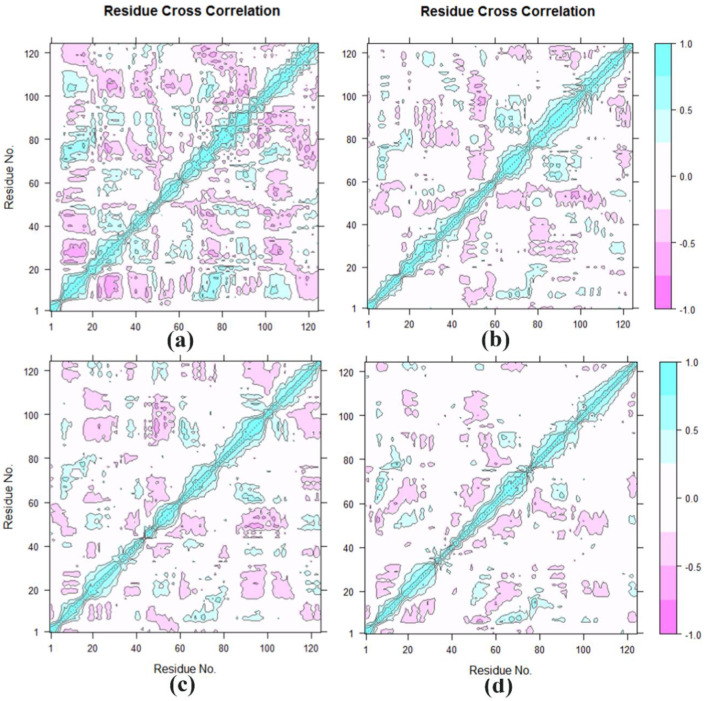
Cross-correlation matrix of the fluctuations of each of the x, y and z coordinates of the Cα atoms from their average during 150 ns MD for (**a**) the Free-BRD4 with the prosthetic group, (**b**)BRD4-Cpd4, (**c**)BRD4-Cpd9, and (**d**) BRD4-Cpd19.

The DCCM for all pairs of Cα atoms was shown in Figure 6a–d. Seen from Figure 6d, it can be concluded that the BRD4-Cpd19 system has the lightest color on the cross-correlation matrix map, indicating that the BRD4-Cpd19 system has experienced the weakest flexibility and the most stability during the MD simulation, which showed that BRD4 was an excellent inhibitor binding to BRD4 with the experimental data (IC_50_: 5.3 ± 0.4 nM) [12].

**Figure 7:**
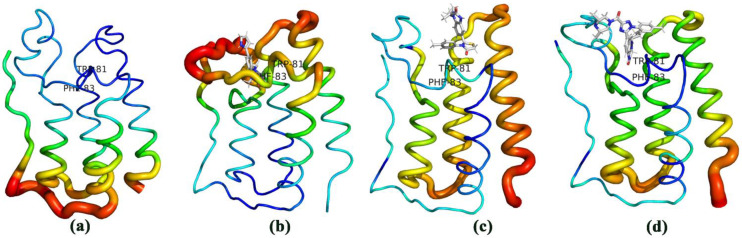
Motions of the four systems (**a**) Free-BRD4, (**b**) BRD4-Cpd4, (**c**) BRD4-Cpd9, and (**d**) BRD4-Cpd19. A thick red line represents a high B value and a thin blue line represents a low B value.

Figure 7a–d showed the B factor value during MD simulations. It was found that the protease catalytic active sites were usually located in the low B value region, Figure 7a is consistent with the conclusion. B factor reflects protein flexibility. The higher the B factor in the protein structure is, the better the mobility of the protein. It can be seen in Figure 7d, the active region of the BRD4-Cpd19 system experienced the weakest flexibility and the most stability during the MD simulation.

**Figure 8:**
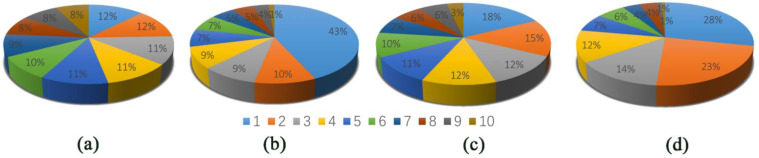
The cluster analysis for (**a**) Free-BRD4, (**b**) BRD4-Cpd4, (**c**) BRD4-Cpd9 and (**d**) BRD4-Cpd19.

In this study, the similar structures of the trajectories of the four systems were divided into different groups using the RMSD-based clustering method (Figure 8a–d). Through cluster analysis, the most representative structure was discovered and was chosen to compare the binding affinities of different ligands. 

**Figure 9:**
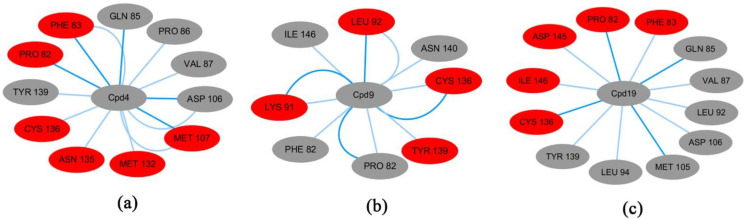
Subnetwork analysis of the protein−ligand interaction. (**a**) Subnetwork betweenBRD4 and Cpd4; (**b**) subnetwork between BRD4 and Cpd9; (**c**) subnetwork between BRD4 and Cpd19.

For BRD4-Cpd4 complex (Figure 9a), Pro82, Phe83, Gln85, Val87, Pro86, Asp106, Met107, Met132, Gln135, Cys136 and Tyr139 had interactions with Cpd4. For BRD4-Cpd9 (Figure 9b), it can be seen that Phe83, Pro82, Lys91, Leu92, Tyr139, Cys136, Ile146 and Asn140 had interactions with Cpd9. From Figure 9c, it can be seen that Pro82, Phe83, Gln85, Val87, Leu92, Leu94, Met105, Asp106, Cys136, Tyr139, Ile146 and Asp145 had interactions with Cpd19. Among three systems, BRD4 is bound most closely to Cpd19 (12 residues), and weakly bound to Cpd4 (11 residues) and Cpd9 (eight residues).

### 3.5. The Free Energy Binding of Three Systems

The free energy between BRD4 and the three inhibitors was calculated in AmberTools17 package [41]. The results were listed in Table 3. In Table 3, the energy in the BRD4-Cpd19 system was lower than that in the two complexes. The results were consistent with experimental data [12].

**Figure 10:**
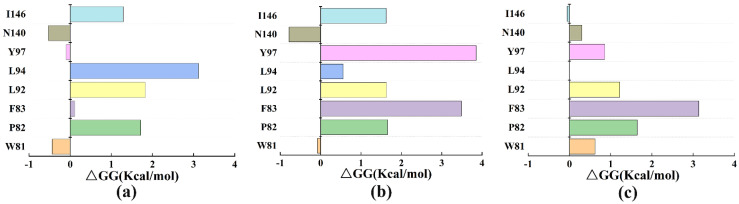
Computational alanine scanning of the active site residues in three complexes. (**a**) BRD4-Cpd4; (**b**) BRD4-Cpd9; (**c**) BRD4-Cpd19.

In this analysis, the residues surrounding the inhibitors (Cpd4, Cpd9 and Cpd19) were mutated to alanine to explore their influence on the stability of protein structures. The results were presented in Figure 10a–c. Among them (Figure 10b,c), mutations in Phe83 indicated the greatest effects on the protein-inhibitor complex, indicating that Phe83 may play crucial roles in binding to Cpd9 and Cpd19. 

## 4. Conclusions

The addition of different inhibitors had different local effects on the protein. Molecular dynamics simulations showed that the binding of three inhibitors influenced the secondary structure changes in BRD4. Only Cpd19 binding to BRD4 induced residues Trp81-Ala89 to partly become a helix but the effects of Cpd4 and Cpd9 were not significant. MM-GBSA calculations suggested that, among three inhibitors, Cpd19 had the lowest binding energy with BRD4 followed by Cpd4, Cpd9.

The above results showed that the Cpd19 combined with BRD4 is the best and consisted with the highly efficient experiment results. Cpd4 and Cpd9, as mild inhibitors, had little effect on the motions of protein and lower binding energy to protein. Residue Trp81-Ala89 in the computer-simulated inhibitor-protein complex may provide some useful clues for further BCPs family search.

## Figures and Tables

**Table 1:** Four systems in 150 ns molecular dynamics simulations.

System	Protein	Ligand
Free-BRD4-BD1	BRD4-BD1	None
BRD4-BD1-Cpd4	BRD4-BD1	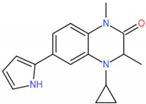
BRD4-BD1-Cpd9	BRD4-BD1	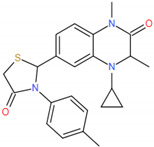
BRD4-BD1-Cpd19	BRD4-BD1	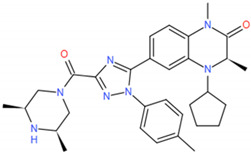

**Table 2:** The probability of the α-helix in the four systems.

Residue No.	Free-BRD4	BRD4-Cpd4	BRD4-Cpd9	BRD4-Cpd19
W81	14.94	14.09	13.20	33.25
P82	14.94	14.09	13.20	33.25
F83	14.94	14.09	13.20	33.25

**Table 3:** MM-GBSA results (kcal/mol).

System	BRD4-Cpd4	BRD4-Cpd9	BRD4-Cpd19
△Gvdw	−32.94 ± 5.14	−38.17 ± 4.10	−46.04 ± 6.40
△Gele	−12.47 ± 6.03	−13.72 ± 5.40	−9.58 ± 8.74
△Gpolar	24.22 ± 4.87	27.73 ± 4.97	28.64 ± 8.62
△Gnonpolar	−4.12 ± 0.48	−4.69 ± 0.47	−5.48 ± 0.76
△Ggas	−45.41 ± 9.18	−51.89 ± 7.81	−55.62 ± 11.58
△Gsolv	20.11 ± 4.63	23.04 ± 4.74	23.15 ± 8.40
△Gtotal	−25.31 ± 5.48	−28.85 ± 4.23	−32.46 ± 5.60
IC_50_	303.0 nM	142.0 ± 3.0 nM	5.3 ± 0.4 nM

## Data Availability

Not applicable.

## Supplementary Materials

The following are available online. Figure S1: (a) Root-mean-square deviation (RMSD) for the backbone atoms, (b) Radius-of-gyration (Rg) plot, (c) Solvent-accessible-surface-area (SASA) plot of the second MD simulation; Figure S2: (a) Root-mean-square deviation (RMSD) for the backbone atoms, (b) Radius-of-gyration (Rg) plot, (c) Solvent-accessible-surface-area (SASA) plot of the third time MD simulation; Figure S3: (a) average Root-mean-square deviation (RMSD) for the backbone atoms, (b) average Radius-of-gyration (Rg) plot of the third time MD simulation, (c) average Solvent-accessible-surface-area (SASA) plot; Figure S4: Binging pocket of The active residues around Cpd 4 binding to BRD4; Figure S5: Cpd 4 redocked to the pocket of BRD4 with AutoDock 4.2.6 software to compared with the docking pose of 6JI3. (blue is Cpd4 of 6JI3 and green is redock Cpd4).
