# Supplementary material for: Molecular Dynamic Simulations of Bromodomain and Extra-Terminal Protein 4 Bonded to Potent Inhibitors
Source: Molecules. 2021 Dec 26;27(1):118. doi: 10.3390/molecules27010118 (PMC8747027; doi:10.3390/molecules27010118)
Supplement: Supplementary file 1 [file molecules-27-00118-s001.zip › molecules-1470600-supplementary.pdf]

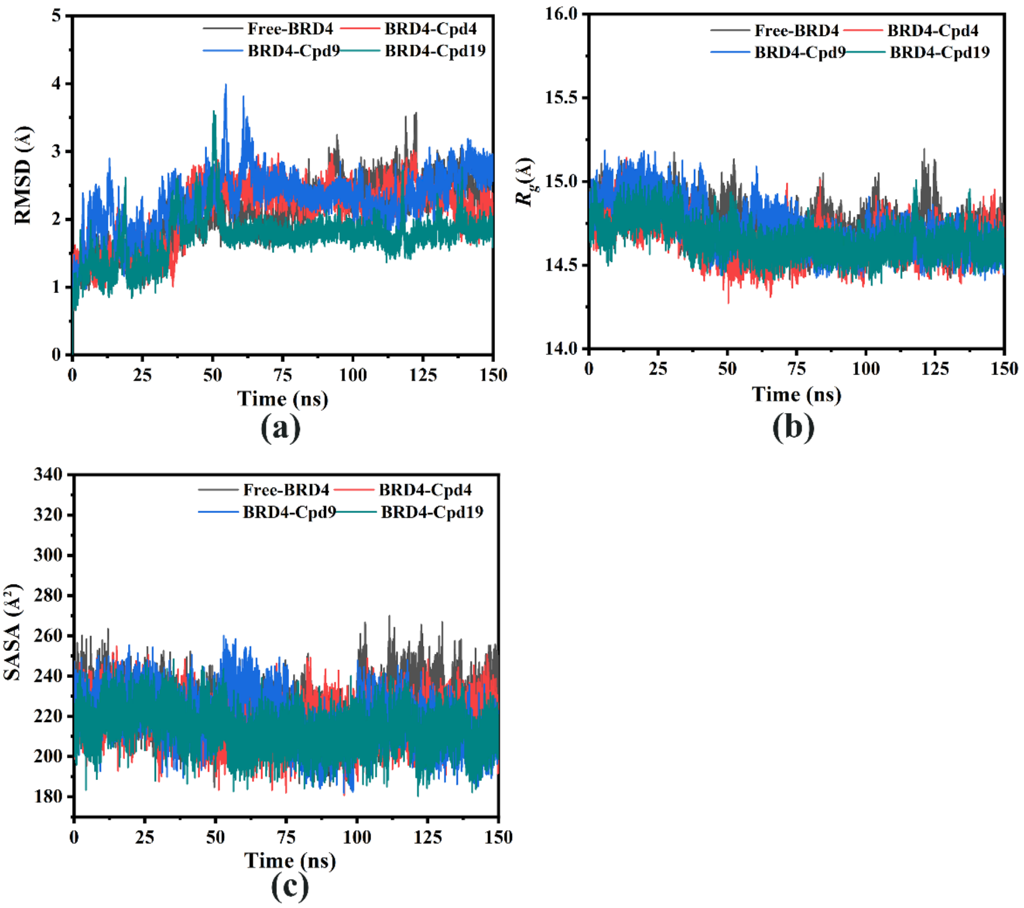

Figure S1. (a) Root-mean-square deviation (RMSD) for the backbone atoms, (b) Radius-of-gyration (R_g) plot, (c) Solvent-accessible-surface-area (SASA) plot of the second MD simulation

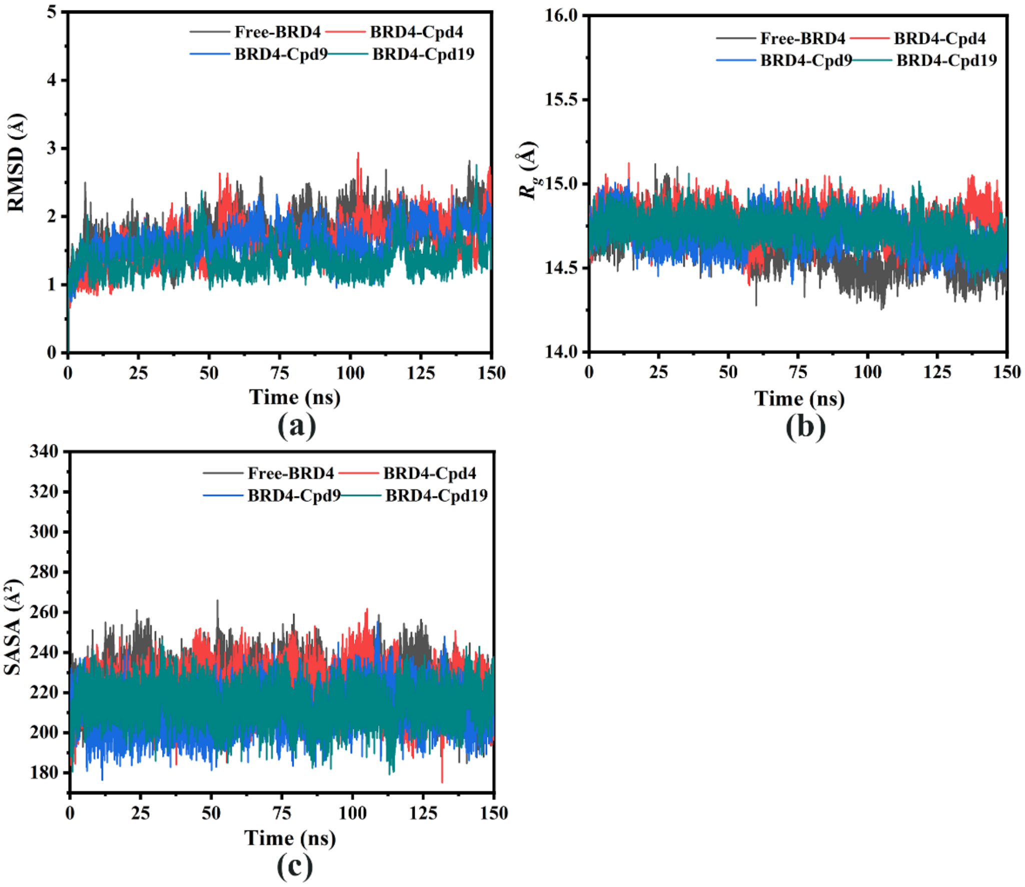

Figure S2. (a) Root-mean-square deviation (RMSD) for the backbone atoms, (b) Radius-of-gyration (R_g) plot, (c) Solvent-accessible-surface-area (SASA) plot of the third time MD simulation.

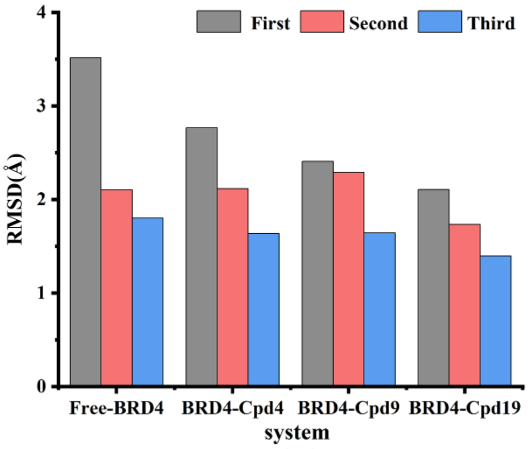

(a)

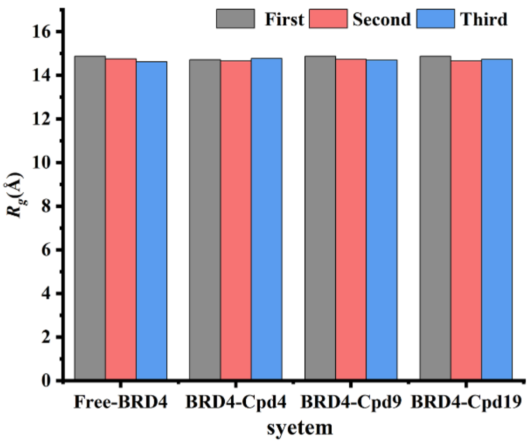

(b)

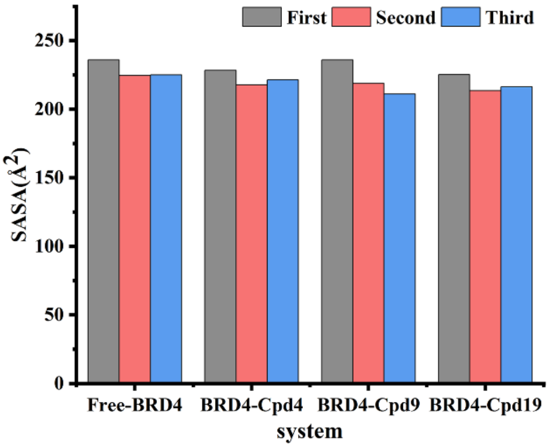

(c)

Figure S3. (a) average Root-mean-square deviation (RMSD) for the backbone atoms, (b) average Radius-of-gyration (R_g) plot of the third time MD simulation, (c) average Solvent-accessible-surface-area (SASA) plot.

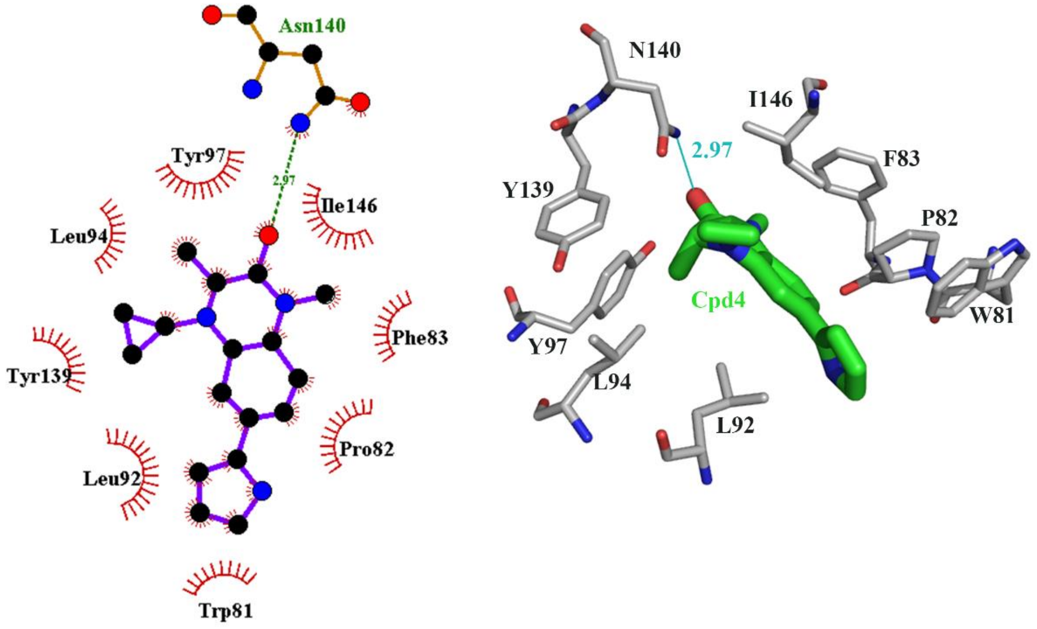

Figure S4. Binging pocket of The active residues around Cpd 4 binding to BRD4

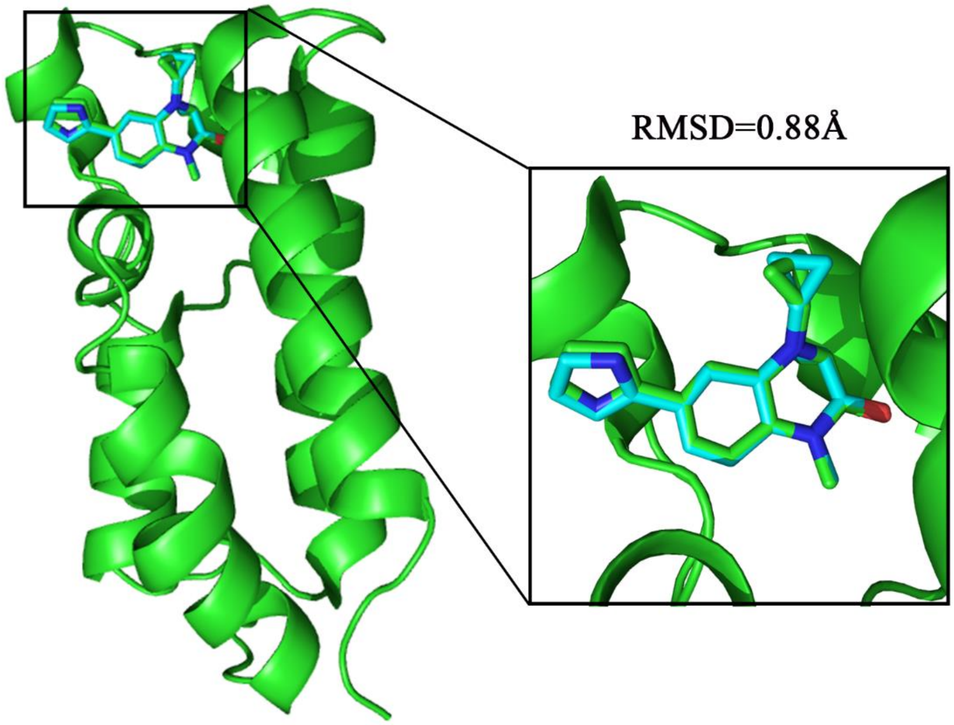

Figure S5. Cpd 4 redocked to the pocket of BRD4 with AutoDock 4.2.6 software to compared with the docking pose of 6JI3. (blue is Cpd4 of 6JI3 and green is redock Cpd4)
